# Albuminuria enhances NHE3 and NCC via stimulation of mitochondrial oxidative stress/angiotensin II axis

**DOI:** 10.18632/oncotarget.9972

**Published:** 2016-06-13

**Authors:** Zhanjun Jia, Yibo Zhuang, Caiyu Hu, Xintong Zhang, Guixia Ding, Yue Zhang, Rajeev Rohatgi, Hu Hua, Songming Huang, John Ci-jiang He, Aihua Zhang

**Affiliations:** ^1^ Department of Nephrology, Nanjing Children's Hospital, Affiliated with Nanjing Medical University, Nanjing, China; ^2^ Jiangsu Key Laboratory of Pediatrics, Nanjing Medical University, Nanjing, China; ^3^ The First Clinical Medical College of Nanjing Medical University, Nanjing, China; ^4^ Department of Medicine, Mount Sinai School of Medicine, New York, New York, USA; ^5^ Division of Nephrology, Department of Medicine, Mount Sinai School of Medicine, New York, New York, USA

**Keywords:** albuminuria, NHE3, NCC, mitochondrial oxidative stress, angiotensin II

## Abstract

Imbalance of salt and water is a frequent and challenging complication of kidney disease, whose pathogenic mechanisms remain elusive. Employing an albumin overload mouse model, we discovered that albuminuria enhanced the expression of NHE3 and NCC but not other transporters in murine kidney in line with the stimulation of angiotensinogen (AGT)/angiotensin converting enzyme (ACE)/angiotensin (Ang) II cascade. In primary cultures of renal tubular cells, albumin directly stimulated AGT/ACE/Ang II and upregulated NHE3 and NCC expression. Blocking Ang II production with an ACE inhibitor normalized the upregulation of NHE3 and NCC in cells. Interestingly, albumin overload significantly reduced mitochondrial superoxide dismutase (SOD2), and administration of a SOD2 mimic (MnTBAP) normalized the expression of NHE3, NCC, and the components of AGT/ACE pathway affected by albuminuria, indicating a key role of mitochondria-derived oxidative stress in modulating renin-angiotensin system (RAS) and renal sodium transporters. In addition, the functional data showing the reduced urinary excretion of Na and Cl and enhanced response to specific NCC inhibitor further supported the regulatory results of sodium transporters following albumin overload. More importantly, the upregulation of NHE3 and NCC and activation of ACE/Ang II signaling pathway were also observed in albuminuric patient kidneys, suggesting that our animal model accurately replicates the human condition. Taken together, these novel findings demonstrated that albuminuria is of importance in resetting renal salt handling via mitochondrial oxidative stress-initiated stimulation of ACE/Ang II cascade. This may also offer novel, effective therapeutic targets for dealing with salt and water imbalance in proteinuric renal diseases.

## INTRODUCTION

In kidney disease, disordered salt and water handling is a common clinical complication. In clinic, a number of patients with nephrotic syndrome (NS) often exhibit salt and water retention. In the past decades, though substantial progress has been made in understanding the kidney role in the regulation of the fluid metabolism, the pathogenic mechanism of fluid retention in NS patients remains unclear. Importantly, recent findings highly suggest that intra-renal autocrine/paracrine factors may contribute to the occurrence of fluid retention observed in NS. Since albuminuria is a common feature of kidney disease, we hypothesized that albuminuria is an important insult resulting in disordered sodium (Na) and water metabolism in albuminuric renal diseases.

It is well known that albuminuria is not only a hallmark of many types of kidney diseases but also an independent contributor of kidney injury [1–3]. Accumulating evidence suggested an important role of oxidative stress in promoting renal reabsorption of salt and water [4–7]. Recently, reports also demonstrated that albuminuria is a potent stimulus of mitochondrial dysfunction leading to the oxidative injury in kidney [8–10]. Therefore, it is reasonable to speculate that mitochondrial oxidative stress might participate in the occurrence of salt and water retention at kidney region.

Systemic and intra-renal renin, angiotensin II (Ang II), and aldosterone system (RAAS) regulate renal sodium transporters [11–17] along the nephron. Specifically, Ang II and aldosterone stimulate the activity and abundance of Na-H exchanger-3 (NHE3) and Na-Cl cotransporter (NCC) [13–17], implying enhanced proximal and distal Na transport. Luminal incubation of albumin with renal tubular cells induces Ang II release suggesting that the presence of albumin can activate the intra-renal RAAS [18]. Moreover, oxidative stress has been shown to be attributable to the stimulation of RAAS [5, 19, 20]. Utilizing animal and cell models and human specimens, we tested whether albuminuria effectively altered Na transporters along the nephron through intra-renal activation of the mitochondrial oxidative stress-RAAS pathway. In details, we investigated: 1) Whether albuminuria could directly upregulate renal sodium transporters. 2) Whether albuminuria-induced mitochondrial oxidative stress could contribute to the dysregulation of renal sodium transporters. 3) Whether RAAS served as a downstream signaling of mitochondrial oxidative stress in modulating renal sodium transporter under albuminuric condition.

## RESULTS

### Effect of albumin overload on the alteration of the renal sodium channel profile induced by albumin overload

**Figure 1:**
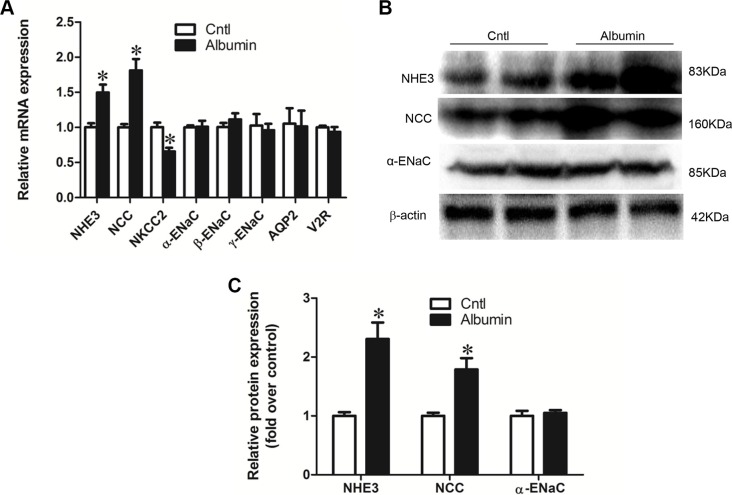
Effects of albuminuria on sodium transporters (**A**) qRT-PCR analyses of NHE3, NCC, NKCC2, α-ENaC, β-ENaC, γ-ENaC, AQP2, and the V2 receptor. (**B**) Western blots of NHE3, NCC, and α-ENaC demonstrate that changes in mRNA expression are recapitulated in transporter protein expression. (**C**) Densitometric analyses confirm that albuminuria enhances NHE3, NCC, and α-ENaC expression. The values represent the means ± SDs (*n* = 6 in each group). *p < 0.01 vs. control mice.

qRT-PCR analysis showed that NHE3 and NCC mRNA levels were significantly induced following albumin overload for 11 days contrasting to a 33% reduction of NKCC2 in mice (Figure 1A). The mRNA expression of the ENaC subunits, AQP2, and V2R, were not affected by albumin overload (Figure 1A). The protein levels of NHE3, NCC, and ENaCα were further confirmed by western blotting (Figure 1B and 1C).

### Diuretic responses to hydrochlorothiazide in albumin-overloaded mice

**Figure 2:**
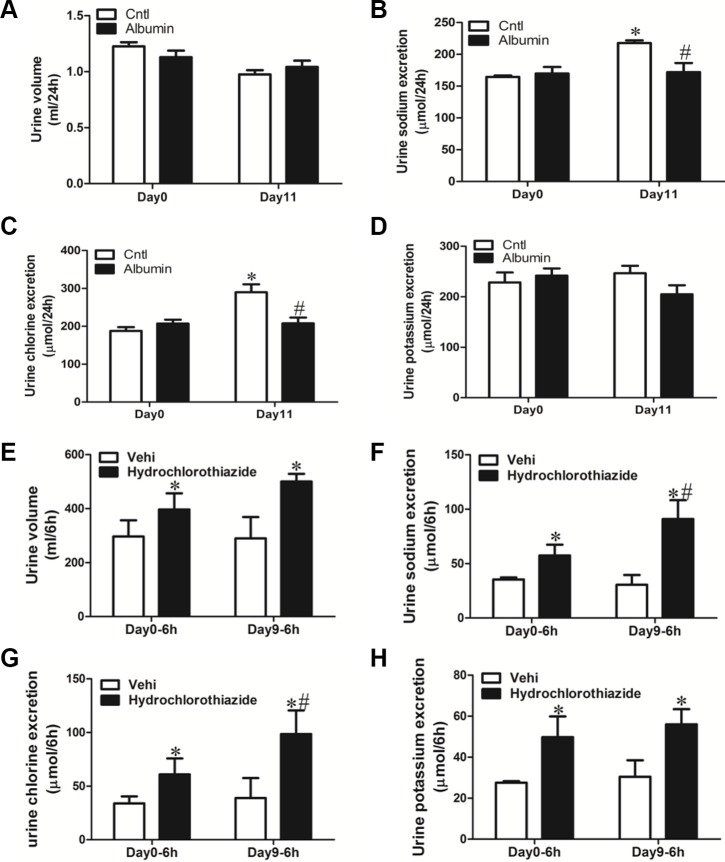
Physiologic study to test the diuretic response of albumin-overloaded mice to NCC inhibitor hydrochlorothiazide (H) (**A–D**) Urine volume and urinary electrolyte excretion in albumin-overloaded mice. (A) Urine volume. (B) Urinary sodium excretion. (C) Urinary chloride excretion. (D) Urinary potassium excretion. (**E–H**) In a separate experiment, the mice were treated with either saline or albumin for up to 9 days and their diuretic response to vehicle (V) or hydrochlorothiazide was measured over 6 h on day 0 and day 9. (E) Urine volume, (F) urinary sodium excretion, (G) urinary chloride excretion, and (H) urinary potassium excretion. The values represent the means ± SDs (*n* = 6 in each group). In Figure 2B and 2C, *p < 0.01 vs. control mice without albumin overload on day 0. ^#^*p* < 0.01 vs. control mice with vehicle (saline) treatment on day 11. In Figure 2E–2F, *p < 0.01 vs. vehicle-treated mice without albumin overload (day 0); ^#^*p* < 0.01 vs. hydrochlorothiazide-treated mice without albumin overload (day 0).

Following albumin overload or vehicle (saline) treatment, mice exhibited reduced urinary output of sodium and chloride (Figure 2A–2D). Meantime, the vehicle-treated mice showed a slightly elevated urinary sodium and chloride excretion as compared with day 0 possibly due to the saline injection (Figure 2B and 2C). To further study the functional role of NCC induction in mice exposed to albumin overload, the NCC inhibitor hydrochlorothiazide (10 mg/kg body weight), was administered to WT mice via i.p injection after albumin overload, and urine was collected for 6 h and analyzed. In line with the induction of NCC expression, the diuretic and natriuretic responses to hydrochlorothiazide were significantly promoted (Figure 2E–2H).

### Blocking Ang II production inhibited the effect of albumin on NHE3 and NCC upregulation

**Figure 3:**
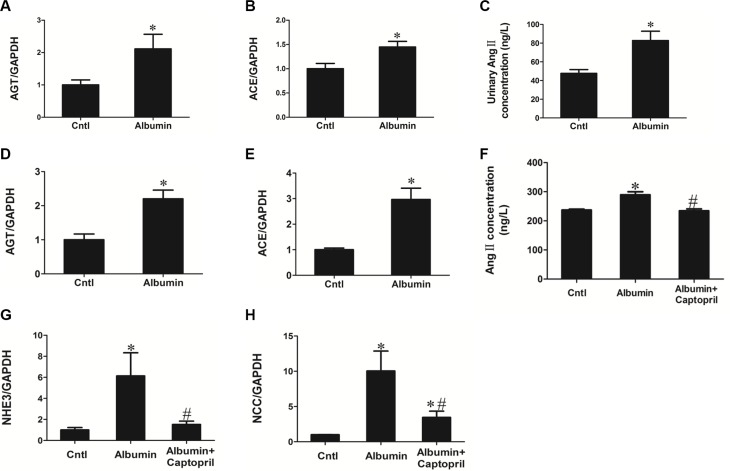
Role of renal RAAS in the albuminuria-induced upregulation of NHE3 and NCC (**A** and **B**) qRT-PCR analyses of AGT (A) and ACE (B) in the kidneys of vehicle-treated mice without albumin and albumin-overloaded mice. (**C**) Urinary Ang II increased in albumin-loaded mice. (**D**) mRNA expression of AGT is induced in primary cultures of tubular cells treated with albumin. (**E**) mRNA expression of ACE is increased in primary cultures of tubular cells treated with albumin. (**F**) Captopril blunted the albumin-induced Ang II production. (**G**) Captopril blocked the albumin-induced upregulation of NHE3, as determined by qRT-PCR. (**H**) Captopril blocked the albumin-induced upregulation of NCC, as determined by qRT-PCR. The values represent the means ± SDs (*n* = 6 in each group). *p < 0.01 vs. control group. ^#^*p* < 0.01 vs. albumin-treated group.

In consideration of the known role of Ang II in regulating renal sodium transporters, we examined Ang II-generating cascade in animals with albumin overload. As expected, albumin overload stimulated the AGT/ACE/Ang II axis in mice, as determined by qRT-PCR in tissue and ELISA in the urine (Figure 3A–3C). In primary culture renal tubular cells, albumin significantly increased AGT and ACE mRNA levels (Figure 3D and 3E) and Ang II secretion in medium (Figure 3F). Additionally, NHE3 and NCC mRNA expression was markedly induced as determined by qRT-PCR (Figure 3G–3I) Following blocking Ang II production with the ACE inhibitor captopril, the induction of NHE3 and NCC was remarkably blunted (Figure 3F–3H). These data indicate a direct effect of albumin on re-setting NHE3, NCC, and demonstrate a key role of renal RAAS in mediating the upregulation of NHE3 and NCC.

### Inhibition of mitochondrial oxidative stress reversed albuminuria-induced abnormalities in sodium transporters and the involved signaling pathways

**Figure 4:**
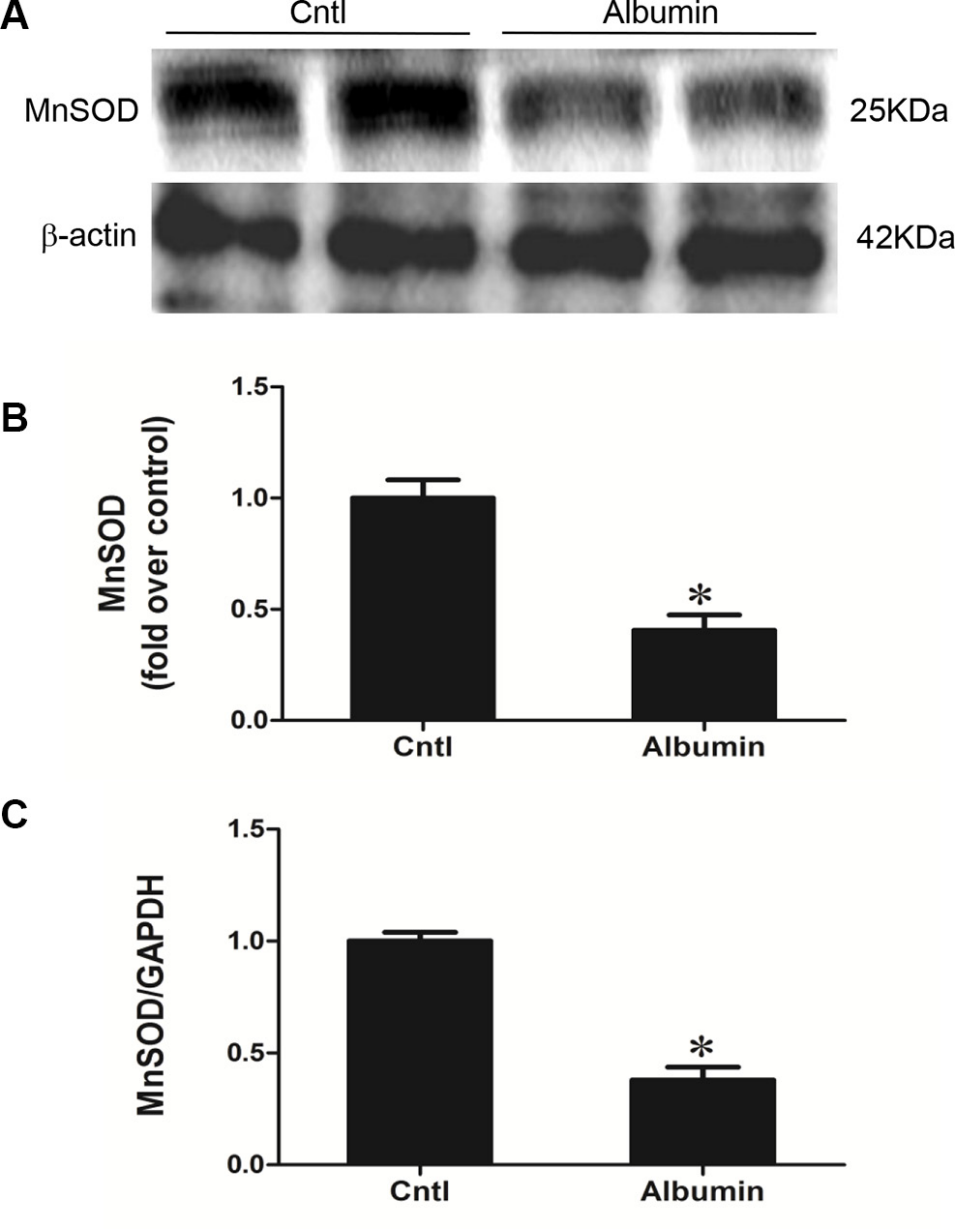
Effects of albumin overload on the regulation of MnSOD (**A**) Western blots of MnSOD. (**B**) Densitometric analysis of SOD2. (**C**) qRT-PCR analysis of SOD2. The values represent the means ± SDs; *n* = 6 in each group. *p < 0.01 vs. control group. ^#^*p* < 0.01 vs. albumin-overloaded group.

**Figure 5:**
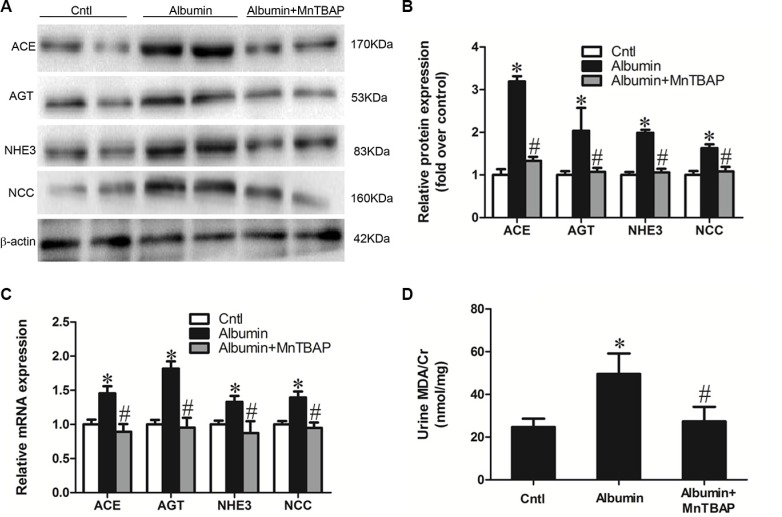
MnTBAP treatment reversed the albuminuria-induced alteration of sodium transporters and their involved signaling pathway (**A**) Western blots of ACE, AGT, NHE3, and NCC illustrates the normalization of protein expression by MnTBAP treatment. (**B**) Densitometric analyses of ACE, AGT NHE3, and NCC. (**C**) qRT-PCR analyses of ACE, AGT, NHE3, and NCC confirm the impression that is observed in the western blot. (**D**) Urinary output of MDA. The values represent the means ± SDs (*n* = 6 in each group). *p < 0.01 vs. control group. ^#^*p* < 0.01 vs. albumin-overloaded group.

Our previous study demonstrated impaired mitochondrial function and enhanced oxidative stress following albumin overload in mice [9]. Here, we further detected a remarkable reduction of mitochondrial superoxide dismutase (MnSOD) in the kidneys of albumin-treated mice (Figure 4A–4C). Application of the mitochondrial SOD mimic MnTBAP completely normalized the expression of NHE3, NCC, and AGT/ACE axis in line with the blockade of albuminuria-induced oxidative stress (Figure 5A–5D). These data indicate that mitochondrial ROS is the initial stimulus of the AGT/ACE/Ang II axis which mediates the effects of albuminuria on renal sodium transporter expression.

### Albuminuric patients have increased ACE/Ang II signaling and altered NHE3 and NCC expression

**Figure 6:**
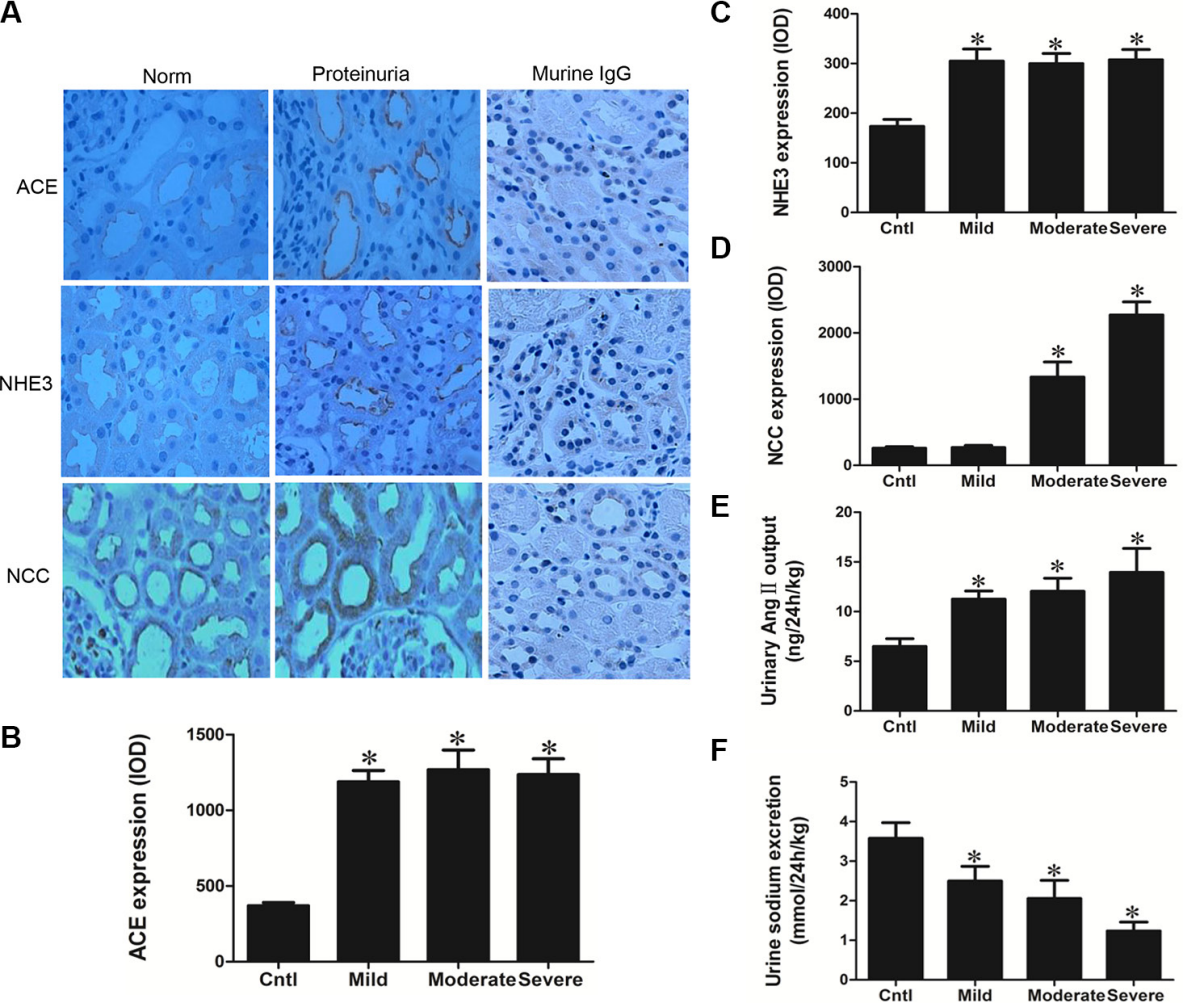
Analyses of ACE, NHE3, NCC, Ang II, and urinary sodium excretion in the proteinuric patients (**A**) Immunohistochemistry of ACE, NHE3, and NCC demonstrate enhanced tubular expression of these elements in proteinuric kidney. (**B**) Semi-quantitative measurement of ACE immunoreactivity. (**C**) Semi-quantitative measurement of NHE3 immunoreactivity. (**D**) Semi-quantitative measurement of NCC immunoreactivity. (**E**) Urinary Ang II excretion was stimulated in proteinuric patients. (**F**) Urinary sodium excretion was reduced in proteinuric patients. The values represent the means ± SDs. For urinary Ang II assay, *n* = 12–33 in each group. In other experiments, *n* = 6 in each group. *p < 0.01 vs. control group.

Kidney biopsy specimens from patients with primary glomerular disease who presented with obvious proteinuria without reaching the diagnostic standard of nephrotic syndrome (plasma albumin no less than 30 g/l) were chosen for the analysis to avoid the influence of blood volume depletion cause by fluid redistribution. As shown by the data, proteinuric patients showed remarkably elevated ACE expression and urinary Ang II excretion (Figure 6A, 6B, and 6E) in line with the significantly enhanced NHE3 and NCC (Figure 6C and 6D). Moreover, the urine sodium excretion was significantly decreased in proteinuric patients compared with normal subjects (Figure 6F), which was largely similar to the phenomenon observed in albumin-overloaded mice. These findings confirm that similar pathways are altered in albuminuric patients and animals, implying that the animal model, at least in part, recapitulates the human condition, a high association between the proteinuric patients and animals in the dysregulation of sodium transporters and the dysfunction of renal fluid handling. And these similar abnormalities in salt handling observed in patients and animals might share the similar pathogenic mechanisms.

## DISCUSSION

Na and water retention are common clinical features of kidney disease that lead to edema, hypertension, and congestive heart failure. The mechanisms underlying Na avidity in albuminuric renal disease is incompletely understood. The principal hypotheses of Na retention in nephrosis has been based on either the “underfill” or “overfill” theory of Na retention. In the “underfill” theory, the assumption is that lower oncotic forces due to hypoalbuminia lead to Na and water leak into the interstitium, intravascular volume depletion that, in turn, activates the RAAS system to induce renal Na retention. However, several lines of evidence suggest that this may not be entirely correct such as (1) albuminuric mice do not develop Na retention, (2) the RAAS system is not consistently activated in nephrosis and (3) raising the oncotic pressure with albumin does not enhance diuresis. The other line of inquiry suggests that Na retention is the primary effect of albuminuria, and hence, the “overfill” theory. Several investigators have suggested that ENaC, expressed in the renal collecting duct, is overexpressed and activated by serine proteases contained in nephrotic urine to induce Na retention [21–24]. The data presented in this manuscript further add evidence that primary Na retention is the culprit pathway leading to extracellular volume expansion in NS, and, that, importantly, urinary albumin itself induces these changes to transporter expression. The major finding of this paper is that albuminuria stimulates the mitochondrial oxidative stress to activate intra-renal RAAS that leads to the upregulation of NCC/NHE3. These findings were corroborated utilizing several methods (molecular and physiologic) and models (primary renal epithelial cell culture, murine kidney, and human kidney) to strengthen their validity. In agreement with our findings, several studies demonstrated the induction of renal NHE3 and RAAS in the proteinuric models [25–27].

We suspect the changes observed in transporter expression contribute to primary Na retention observed in NS. In particular, the upregulation of NCC, which was demonstrated at a molecular (mRNA, protein, immunohistochemistry) and physiologic (heightened diuresis related to thiazide) level, implies the contribution of NCC to Na retention. Recent evidence demonstrates the importance of the ENaC to Na avidity in NS which, in light of this new data, suggests the importance of the distal nephron in Na retention. In this work we did not observe a change in ENaC subunit expression; however, this may reflect differences in the models studied (puromycin induced nephrosis vs. albumin overload) [28, 29]. Moreover, our model does not induce generalized proteinuria, commonly observed in NS, which may be the source of serine proteases that cleave and activate ENaC to induce ENaC dependent Na absorption. The fact that albumin-induced oxidative stress stimulates the effects on Na transporter expression and that suppressing oxidative stress restores transporter expression suggests that oxidative stress may be an important future therapeutic target to normalize Na balance in albuminuric renal disease. Consistent with the restoration of the elevated NHE3 and NCC following MnTBAP therapy in this study, the evidence from our and other groups also showed that inhibition of mitochondrial ROS production or RAAS activation induced by albumin overload attenuated tubular damage [30, 31].

The limitation of this study is that the animal model we used is not an exact mimic of a glomerular disease-related proteinuria, as is normally observed with NS. Also the current model had no the features of low oncotic pressure and edema presented by NS. However, this unique model largely avoided the effects of renal failure and severe alteration of renal hemodynamic besides albuminuria itself and permitted us to clearly study the actions of urinary albumin on renal transporter expression. In summary, our studies, validated in mouse, human, and *in vitro* cells, demonstrate an important role of albuminuria in modulating renal Na transporter expression and Na retention via stimulating mitochondrial oxidative stress/RAS signaling pathway. These novel findings not only advance our understanding of how albuminuric renal disease leads to excess extracellular fluid volume, but also shed new light on the development of new therapeutic strategies.

## MATERIALS AND METHODS

### Animals

C57BL/6J mice were originally purchased from the Jackson laboratory. This mouse colony was propagated at Nanjing Medical University. In all experiments, 3- to 4-mo-old male mice were used. All of the mice were maintained under a 12:12-h light-dark cycle (lights on at 6:00 a.m. and lights off at 6:00 p.m.). Animal studies were performed under protocols in accordance with relevant guidelines and regulations and approved by the Nanjing Medical University Institutional Animal Care and Use Committee (No. 20090053).

### Reagents and antibodies

DMEM-F12, newborn bovine serum, HEPES and penicillin/streptomycin were purchased from Wisent Corporation (Wisent, Canada). Hank's balanced salt solution (HBSS), nonessential amino acids, sodium pyruvate, insulin-transferrin-selenium and L-glutamine were purchased from Invitrogen Life Technologies (Paisley, Scotland). Stainless steel sieves were obtained from Merck Eurolab (Leuven, Belgium). Anti-NCC (Stressmarq Biosciences Inc., Canada), anti-NHE3 (Abcam, Cambridge, MA), anti-AGT (Abcam, Cambridge, MA), anti-ACE (Santa Cruz Biotechnology, Santa Cruz, CA), and anti-MnSOD (Santa Cruz Biotechnology, Santa Cruz, CA) primary antibodies and horseradish peroxidase (HRP)-conjugated secondary antibodies (Santa Cruz Biotechnology, Santa Cruz, CA) were used.

### Human renal biopsy specimens

Renal biopsy samples were obtained from patients undergoing diagnostic evaluation at the Department of Nephrology of Nanjing Children's Hospital, which is affiliated with Nanjing Medical University. Eighteen subjects (age range: 2–15 years old) were selected based on the criterion of having at least ten glomeruli in a paraffin-embedded tissue sample available for histological sectioning. All biopsy specimens were evaluated by a pathologist who was unaware of the results of the molecular studies. The samples were divided into the following categories according to the severity of proteinuria: mild proteinuria (< 1.0 g/24 h, *n* = 6), moderate proteinuria (1.0–3.0 g/24 h, *n* = 6), and severe proteinuria (> 3.0 g/24 h, *n* = 6). Normal renal tissues were obtained from patients without proteinuria who underwent partial nephrectomy for benign renal tumors. The study was approved by the ethics committee at Nanjing Children's Hospital, China (LL20130432).

### Human urine samples

Urine samples were collected from the patients (age range: 2–15 years old) 24 h after being hospitalized at the Department of Nephrology of Nanjing Children's Hospital. Fifty-six urine samples were collected from patients diagnosed with primary proteinuric kidney diseases but with normal serum albumin levels. These samples were divided into the following categories according to the severity of proteinuria: mild proteinuria (< 1.0 g/24 h, *n* = 26), moderate proteinuria (1.0–3.0 g/24 h, *n* = 12), and severe proteinuria (> 3.0 g/24 h, *n* = 18). Twenty-four-hour normal urine was also obtained from 33 hospitalized patients without proteinuria. Before urine was collected, we obtained permission from the parents of the patients. The urine was promptly centrifuged to remove the sediment and stored at −80°C. Urine osmolality was measured using an osmometer (Osmett II, Precision Systems, Natick, MA), and urine sodium concentration was measured using a flame photometer (Instrumentation Laboratory, Lexington, MA). This study was approved by the ethics committee at Nanjing Children's Hospital, China (201407005-1).

### Albumin overload experiments

Eight-week-old male mice (25–30 g each) received intraperitoneal (IP) injections daily for 11 days with low-endotoxin bovine serum albumin (BSA) (A-9430, Sigma Chemical Co., St. Louis, MO) dissolved in saline. BSA was administered for 5 days using a stepwise, incremental dose regimen, with the doses rising from 2 mg/g body weight on the first day (D1) to a maximum dose of 10 mg/g on the fifth day, which was maintained thereafter for 6 days. In MnTBAP experiment, control group was given a corresponding volume of saline via intraperitoneal injection, and albumin-treated mice received vehicle or MnTBAP (10 mg·kg^−1^·day^−1^) treatment for 11 days. At the termination of the experiments, the mice were anesthetized with an IP injection of a ketamine/xylazine/atropine, and plasma and kidney samples were immediately frozen in liquid nitrogen and stored at −80°C until use.

### Responses to hydrochlorothiazide

NCC inhibitor of hydrochlorothiazide at a dose of 10 mg/kg body weight was given to the WT mice with or without albumin overload by IP injection. Urine was collected for exactly 6 hours, the urine volume was then measured, and the urine sodium, potassium and chloride concentrations were analyzed using a flame photometer (Instrumentation Laboratory, Lexington, MA).

### Primary culture of mouse renal tubular cells

Eight-week-old male mice were anesthetized with an IP injection of a ketamine/xylazine/atropine solution, their aortas were clamped off below the kidneys, and their kidneys were washed with phosphate-buffered saline (PBS). Following complete blood washout, the kidneys were excised, and the renal cortices manually dissected in ice-cold dissection solution (DS) (HBSS with 10 mmol/l glucose, 5 mmol/l glycine, 1 mmol/l alanine, and 15 mmol/l HEPES pH 7.4 at an osmolality of 325 mosmol/kg H_2_O) into 1-mm-wide pieces. The fragments were ground and sieved through two stainless steel sieves (pore sizes 80 microns and 160 microns) in DS, and the tubule fragments obtained from the 160-micron sieve were centrifuged for 5 min at 1000 r/min. After centrifugation, the supernatant was discarded, and 0.05% trypsin with EDTA) added for 20 min at 37°C to digest the tubular fragments. DMEM-F12 containing 10% FBS was used to stop the digestion. The solution containing the tubular fragments was then centrifuged for 5 min at 1000 r/min, and after discarding the supernatant, the tubular cells were resuspended in the appropriate amount of culture medium (DMEM/F12 supplemented with 10% newborn bovine serum, 15 mmol/l HEPES, 2 mmol/l L-glutamine, 50 nmol/l hydrocortisone, 5 g/ml insulin, 5 g/ml transferrin, 50 nmol/l selenium, 0.55 mmol/l sodium pyruvate, 10 ml/l 100X nonessential amino acids, 100 IU/ml penicillin and 100 g/ml streptomycin buffered to pH 7.4 at an osmolality of 325 mosmol/kg H_2_O). The tubular cells were then seeded onto collagen-coated 6-well cell culture plates (Greiner, Germany) and left unstirred for 48 h at 37°C and 95% air-5% CO_2_ in a standard humidified incubator (Thermo, UK), after which the culture medium was changed for the first time. The medium was then replaced every 2 days, and after 5 days, the cells grew into a confluent monolayer. Then, the cells were treated with albumin (10 mg/ml) for 24 h following a 30-min pretreatment with captopril (50 μm/l).

### Quantitative real-time PCR (qRT-PCR)

Total DNA and RNA were extracted using the DNeasy Tissue Kit (Qiagen, Valencia, CA, USA) and TRIzol reagent (Invitrogen), respectively. Oligonucleotides were designed using Primer3 software (available at http://frodo.wi.mit.edu/) and synthesized by Invitrogen. The sequences of the primer pairs are shown in Table 1. qRT-PCR was then used to detect the mRNA expression of the target genes, and reverse transcription was performed using a reaction kit (Promega Reverse Transcription System) according to the manufacturer's protocol. Real-time PCR amplification was performed using the ABI 7500 real-time PCR detection system (Foster City, CA, USA) with the SYBR Green PCR Master Mix (Applied Biosystems). The cycling conditions were 95°C for 10 min, followed by 40 cycles of 95°C for 15 s and 60°C for 1 min. The mRNA levels were normalized to a GAPDH control and calculated using the comparative cycle threshold (ΔΔCt) method.

**Table 1:** Primer sequences for qRT-PCR

Gene symbol	Primer sequences
GAPDH	5′- GTCTTCACTACCATGGAGAAGG -3′
	5′- TCATGGATGACCTTGGCCAG -3′
α-ENaC	5′- GCTTCATCTTTACCTGTCGTTTC -3′
	5′- CCAGAGATTGGAGTTGTTCTTGT -3′
β-ENaC	5′- CAGTGGGGAGTCTTCATCC -3′
	5′- TCCTGGTGGTGTTGCTGT -3′
γ-ENaC	5′- CTGCTTCTTCGATGGGATG -3′
	5′- GACACCAGGAAGGGGTTGT -3′
NCC	5′- GACAGGCACCAACAGTGAGA -3′
	5′- TAGAGATGGCGGAGATGGAG -3′
NKCC2	5′- GCTCTTCATTCGCCTCTCCT -3′
	5′- AGCCTATTGACCCACCGAAC -3′
NHE3	5′- CTGAGGAGGAACCGAGCA -3′
	5′- AGGCCCAGAACGATGAGTAG-3′
AQP2	5′- GGACCTGGCTGTCAATGCT-3′
	5′- ATCGGTGGAGGCAAAGATG-3′
V2R	5′- TCATCAGCCACCACACCA -3′
	5′- AGATAGCAGGGCCAGTTCAG -3′
ACE	5′-TTGCTATGGGCATGGAAGAG-3′
	5′-CAGGTCTTGCTCCAGGTTGT-3′
AGT	5′-TGTGACAGGGTGGAAGATGA-3′
	5′-AGATCATGGGCACAGACACC-3′

### Western blotting

mPTCs were lysed using a protein lysis buffer containing 50 mM Tris, 150 mM NaCl, 10 mM EDTA, 1% Triton X-100, 200 mM sodium fluoride, and 4 mM sodium orthovanadate, as a protease inhibitor (pH 7.5). Immunoblotting was then performed using primary antibodies against NHE3 (1:500), NCC (1:500), ACE (1:500), AGT (1:300), MnSOD (1:300) and β-actin (1:1000), followed by the addition of HRP-labeled secondary antibodies. The blots were then visualized using the Amersham ECL detection system (Amersham, Little Chalfont, UK), and densitometric analysis was performed using Quantity One software (Bio-Rad).

### Immunostaining

Kidneys were fixed in 4% paraformaldehyde, embedded in paraffin and then cut into 3-μm-thick sections (Cryostat 2800 Frigocut-E, Leica Instruments), and a standard protocol using xylene and graded ethanol was employed to deparaffinize and rehydrate the tissues. The sections were washed with PBS and treated with blocking buffer containing 50 mM NH_4_Cl, 2% BSA, and 0.05% saponin in PBS for 20 min at room temperature. The sections were then incubated overnight at 4°C with primary antibodies of anti-NHE3 rabbit polyclonal antibody (1:20), anti-NCC rabbit polyclonal antibody (1:200), and anti-ACE rabbit polyclonal antibody (1:200). After washing with PBS, the secondary antibody was applied, and the signals were visualized using an ABC kit (Santa Cruz Biotechnology, Santa Cruz, CA).

### EIA assay

Urine samples were centrifuged for 5 min at 10,000 rpm. Cell culture supernatants were centrifuged for 15 min at 3,000 rpm. Ang II concentrations were measured using an enzyme-linked immunosorbent assay (ELISA) kit according to the manufacturer's instructions (Abcam, Cambridge, MA).

### Statistical analysis

All data are presented as the means ± standard deviations (SDs). Statistical analysis was performed using ANOVA followed by Bonferroni's test or unpaired Student's test with the SPSS 13 statistical software. *P* < 0.05 was considered significant.
